# 2-Chloro-6-methoxy­quinoline-3-carbaldehyde

**DOI:** 10.1107/S1600536809040847

**Published:** 2009-10-13

**Authors:** R. Subashini, F. Nawaz Khan, Machhindra Gund, Venkatesha R. Hathwar, Seik Weng Ng

**Affiliations:** a Chemistry Division, School of Science and Humanities, VIT University, Vellore 632 014, Tamil Nadu, India; b Solid State and Structural Chemistry Unit, Indian Institute of Science, Bangalore 560 012, Karnataka, India; c Department of Chemistry, University of Malaya, 50603 Kuala Lumpur, Malaysia

## Abstract

The quinoline fused-ring system of the title compound, C_11_H_8_ClNO_2_, is planar (r.m.s. deviation = 0.0095 Å); the formyl group is slightly bent out of this plane [C—C—C—O torsion angles = −2.4 (3) and 175.9 (2)°].

## Related literature

For a review of the synthesis of quinolines by the Vilsmeier–Haack reaction, see: Meth-Cohn (1993 ▶).
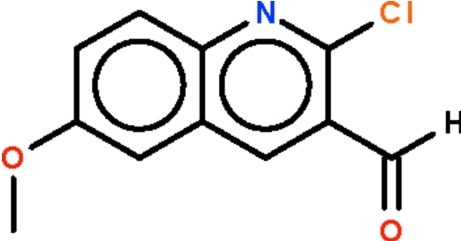


## Experimental

### 

#### Crystal data

C_11_H_8_ClNO_2_
*M* *_r_* = 221.63
Monoclinic, 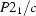

*a* = 7.7072 (9) Å
*b* = 14.3474 (13) Å
*c* = 9.3487 (10) Å
β = 109.415 (2)°
*V* = 974.98 (18) Å^3^
*Z* = 4
Mo *K*α radiation
μ = 0.37 mm^−1^
*T* = 290 K
0.24 × 0.21 × 0.18 mm

#### Data collection

Bruker SMART area-detector diffractometer
Absorption correction: multi-scan (*SADABS*; Sheldrick, 1996 ▶) *T*_min_ = 0.917, *T*_max_ = 0.937
6533 measured reflections
2221 independent reflections
1702 reflections with *I* > 2σ(*I*)
*R*_int_ = 0.030

#### Refinement

*R*[*F* ^2^ > 2σ(*F* ^2^)] = 0.038
*wR*(*F* ^2^) = 0.115
*S* = 1.03
2221 reflections
137 parameters
H-atom parameters constrained
Δρ_max_ = 0.21 e Å^−3^
Δρ_min_ = −0.24 e Å^−3^

### 

Data collection: *SMART* (Bruker, 2004 ▶); cell refinement: *SAINT* (Bruker, 2004 ▶); data reduction: *SAINT*; program(s) used to solve structure: *SHELXS97* (Sheldrick, 2008 ▶); program(s) used to refine structure: *SHELXL97* (Sheldrick, 2008 ▶); molecular graphics: *X-SEED* (Barbour, 2001 ▶); software used to prepare material for publication: *publCIF* (Westrip, 2009 ▶).

## Supplementary Material

Crystal structure: contains datablocks global, I. DOI: 10.1107/S1600536809040847/bt5087sup1.cif

Structure factors: contains datablocks I. DOI: 10.1107/S1600536809040847/bt5087Isup2.hkl

Additional supplementary materials:  crystallographic information; 3D view; checkCIF report

## Figures and Tables

**Fig. 1.**
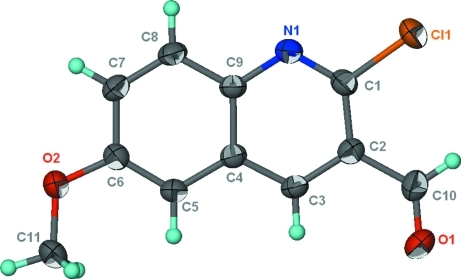
Anisotropic displacement ellipsoid plot (Barbour, 2001) of the title compound at the 50% probability level; hydrogen atoms are drawn as spheres of arbitrary radius.

## supplementary crystallographic information

### Experimental

A Vilsmeier-Haack adduct prepared from phosphorus oxytrichloride (6.5 ml, 70 mmol) and *N*,*N*-dimethylformamide (2.3 ml, 30 mmol) at 273 K was added to *N*-(4-anisyl)acetamide (1.65 g, 10 mmol). The mixture was heated at 353 K for 15 h. The mixture was poured onto ice; the white product was collected and dried. The compound was purified by recrystallization from a petroleum ether/ethyl acetate mixture.

### Refinement

H-atoms were placed in calculated positions (C–H 0.93–0.96 Å) and were included in the refinement in the riding model approximation, with *U*(H) set to 1.2–1.5*U*(C).

### Crystal data

**Table:**

C_11_H_8_ClNO_2_	*F*(000) = 456
*M**_r_* = 221.63	*D*_x_ = 1.510 Mg m^−^^3^
Monoclinic, *P*2_1_/*c*	Mo *K*α radiation, λ = 0.71073 Å
Hall symbol: -P 2ybc	Cell parameters from 842 reflections
*a* = 7.7072 (9) Å	θ = 2.0–24.7°
*b* = 14.3474 (13) Å	µ = 0.37 mm^−^^1^
*c* = 9.3487 (10) Å	*T* = 290 K
β = 109.415 (2)°	Block, colorless
*V* = 974.98 (18) Å^3^	0.24 × 0.21 × 0.18 mm
*Z* = 4	

### Data collection

**Table:**

Bruker SMART area-detector diffractometer	2221 independent reflections
Radiation source: fine-focus sealed tube	1702 reflections with *I* > 2σ(*I*)
graphite	*R*_int_ = 0.030
φ and ω scans	θ_max_ = 27.5°, θ_min_ = 2.7°
Absorption correction: multi-scan (*SADABS*; Sheldrick, 1996)	*h* = −10→9
*T*_min_ = 0.917, *T*_max_ = 0.937	*k* = −18→10
6533 measured reflections	*l* = −12→12

### Refinement

**Table:**

Refinement on *F*^2^	Primary atom site location: structure-invariant direct methods
Least-squares matrix: full	Secondary atom site location: difference Fourier map
*R*[*F*^2^ > 2σ(*F*^2^)] = 0.038	Hydrogen site location: inferred from neighbouring sites
*wR*(*F*^2^) = 0.115	H-atom parameters constrained
*S* = 1.02	*w* = 1/[σ^2^(*F*_o_^2^) + (0.068*P*)^2^ + 0.0419*P*] where *P* = (*F*_o_^2^ + 2*F*_c_^2^)/3
2221 reflections	(Δ/σ)_max_ = 0.001
137 parameters	Δρ_max_ = 0.21 e Å^−^^3^
0 restraints	Δρ_min_ = −0.24 e Å^−^^3^

### Fractional atomic coordinates and isotropic or equivalent isotropic displacement parameters (Å^2^)

**Table:**

	*x*	*y*	*z*	*U*_iso_*/*U*_eq_	
Cl1	0.12213 (7)	0.04921 (3)	0.68956 (6)	0.05816 (19)	
O1	0.02629 (18)	0.34429 (9)	0.71932 (16)	0.0563 (4)	
O2	0.77125 (17)	0.34949 (8)	0.35759 (14)	0.0480 (3)	
N1	0.35512 (19)	0.10458 (9)	0.56310 (15)	0.0399 (3)	
C1	0.2333 (2)	0.13533 (11)	0.62026 (18)	0.0386 (4)	
C2	0.1913 (2)	0.23022 (11)	0.63341 (17)	0.0365 (4)	
C3	0.2892 (2)	0.29435 (11)	0.58240 (18)	0.0366 (3)	
H3	0.2659	0.3576	0.5885	0.044*	
C4	0.4244 (2)	0.26584 (10)	0.52104 (16)	0.0340 (3)	
C5	0.5323 (2)	0.32923 (11)	0.47029 (18)	0.0372 (4)	
H5	0.5165	0.3931	0.4772	0.045*	
C6	0.6601 (2)	0.29554 (11)	0.41088 (18)	0.0375 (4)	
C7	0.6864 (2)	0.19874 (12)	0.40126 (18)	0.0406 (4)	
H7	0.7733	0.1771	0.3600	0.049*	
C8	0.5865 (2)	0.13662 (11)	0.45142 (18)	0.0403 (4)	
H8	0.6067	0.0730	0.4456	0.048*	
C9	0.4520 (2)	0.16836 (10)	0.51239 (17)	0.0342 (3)	
C10	0.0534 (2)	0.26334 (14)	0.70166 (19)	0.0446 (4)	
H10	−0.0153	0.2191	0.7322	0.054*	
C11	0.7544 (3)	0.44805 (12)	0.3677 (2)	0.0542 (5)	
H11A	0.8378	0.4783	0.3259	0.081*	
H11B	0.7835	0.4657	0.4721	0.081*	
H11C	0.6306	0.4665	0.3120	0.081*	

### Atomic displacement parameters (Å^2^)

**Table:**

	*U*^11^	*U*^22^	*U*^33^	*U*^12^	*U*^13^	*U*^23^
Cl1	0.0724 (4)	0.0424 (3)	0.0754 (4)	−0.0099 (2)	0.0455 (3)	0.0028 (2)
O1	0.0572 (8)	0.0491 (9)	0.0728 (9)	0.0069 (6)	0.0355 (7)	−0.0077 (6)
O2	0.0529 (7)	0.0395 (7)	0.0631 (8)	−0.0047 (5)	0.0347 (6)	−0.0021 (5)
N1	0.0497 (8)	0.0302 (8)	0.0442 (7)	0.0003 (6)	0.0216 (6)	−0.0002 (5)
C1	0.0455 (9)	0.0337 (8)	0.0394 (8)	−0.0033 (7)	0.0176 (7)	0.0014 (6)
C2	0.0376 (8)	0.0358 (8)	0.0369 (8)	0.0018 (6)	0.0136 (7)	−0.0017 (6)
C3	0.0423 (8)	0.0285 (8)	0.0411 (8)	0.0043 (6)	0.0168 (7)	−0.0013 (6)
C4	0.0379 (8)	0.0307 (8)	0.0346 (8)	0.0030 (6)	0.0135 (6)	0.0001 (6)
C5	0.0425 (8)	0.0278 (8)	0.0435 (8)	0.0003 (6)	0.0174 (7)	−0.0018 (6)
C6	0.0393 (8)	0.0353 (9)	0.0397 (8)	−0.0029 (6)	0.0157 (7)	−0.0001 (6)
C7	0.0437 (9)	0.0385 (9)	0.0442 (9)	0.0056 (7)	0.0209 (7)	−0.0032 (7)
C8	0.0493 (9)	0.0303 (9)	0.0448 (9)	0.0069 (7)	0.0205 (7)	−0.0013 (6)
C9	0.0406 (8)	0.0278 (8)	0.0350 (8)	0.0019 (6)	0.0138 (6)	−0.0001 (6)
C10	0.0431 (9)	0.0510 (11)	0.0445 (9)	0.0002 (8)	0.0208 (7)	−0.0024 (8)
C11	0.0605 (12)	0.0381 (10)	0.0751 (13)	−0.0109 (8)	0.0373 (10)	−0.0039 (8)

### Geometric parameters (Å, °)

**Table:**

Cl1—C1	1.7461 (16)	C4—C9	1.421 (2)
O1—C10	1.201 (2)	C5—C6	1.370 (2)
O2—C6	1.3654 (18)	C5—H5	0.9300
O2—C11	1.426 (2)	C6—C7	1.411 (2)
N1—C1	1.302 (2)	C7—C8	1.359 (2)
N1—C9	1.362 (2)	C7—H7	0.9300
C1—C2	1.414 (2)	C8—C9	1.414 (2)
C2—C3	1.372 (2)	C8—H8	0.9300
C2—C10	1.487 (2)	C10—H10	0.9300
C3—C4	1.407 (2)	C11—H11A	0.9600
C3—H3	0.9300	C11—H11B	0.9600
C4—C5	1.416 (2)	C11—H11C	0.9600
			
C6—O2—C11	117.12 (13)	C5—C6—C7	120.73 (14)
C1—N1—C9	117.96 (13)	C8—C7—C6	120.92 (14)
N1—C1—C2	125.38 (14)	C8—C7—H7	119.5
N1—C1—Cl1	114.97 (12)	C6—C7—H7	119.5
C2—C1—Cl1	119.62 (12)	C7—C8—C9	120.21 (14)
C3—C2—C1	116.56 (14)	C7—C8—H8	119.9
C3—C2—C10	119.25 (15)	C9—C8—H8	119.9
C1—C2—C10	124.17 (15)	N1—C9—C8	118.96 (14)
C2—C3—C4	120.94 (14)	N1—C9—C4	122.11 (13)
C2—C3—H3	119.5	C8—C9—C4	118.93 (14)
C4—C3—H3	119.5	O1—C10—C2	123.35 (17)
C3—C4—C5	123.14 (14)	O1—C10—H10	118.3
C3—C4—C9	117.05 (14)	C2—C10—H10	118.3
C5—C4—C9	119.81 (13)	O2—C11—H11A	109.5
C6—C5—C4	119.40 (15)	O2—C11—H11B	109.5
C6—C5—H5	120.3	H11A—C11—H11B	109.5
C4—C5—H5	120.3	O2—C11—H11C	109.5
O2—C6—C5	124.81 (15)	H11A—C11—H11C	109.5
O2—C6—C7	114.46 (13)	H11B—C11—H11C	109.5
			
C9—N1—C1—C2	0.5 (2)	C4—C5—C6—C7	−0.6 (2)
C9—N1—C1—Cl1	−177.49 (11)	O2—C6—C7—C8	179.38 (15)
N1—C1—C2—C3	−0.6 (2)	C5—C6—C7—C8	−0.4 (2)
Cl1—C1—C2—C3	177.35 (12)	C6—C7—C8—C9	0.9 (2)
N1—C1—C2—C10	−178.93 (16)	C1—N1—C9—C8	179.30 (15)
Cl1—C1—C2—C10	−1.0 (2)	C1—N1—C9—C4	0.3 (2)
C1—C2—C3—C4	−0.2 (2)	C7—C8—C9—N1	−179.39 (15)
C10—C2—C3—C4	178.22 (15)	C7—C8—C9—C4	−0.4 (2)
C2—C3—C4—C5	−178.40 (15)	C3—C4—C9—N1	−1.1 (2)
C2—C3—C4—C9	1.0 (2)	C5—C4—C9—N1	178.34 (14)
C3—C4—C5—C6	−179.51 (15)	C3—C4—C9—C8	179.98 (14)
C9—C4—C5—C6	1.1 (2)	C5—C4—C9—C8	−0.6 (2)
C11—O2—C6—C5	1.3 (2)	C3—C2—C10—O1	−2.4 (3)
C11—O2—C6—C7	−178.48 (16)	C1—C2—C10—O1	175.9 (2)
C4—C5—C6—O2	179.62 (15)		
